# The relationship between urine heat shock protein 70 and congenital anomalies of the kidney and urinary tract: UTILISE study

**DOI:** 10.3389/fruro.2023.1281081

**Published:** 2024-02-28

**Authors:** Bagdagul Aksu, Alberto Caldas Afonso, Ipek Akil, Harika Alpay, Bahriye Atmis, Ozlem Aydog, Sevcan Bakkaloglu, Aysun Karabay Bayazıt, Meral Torun Bayram, Ilmay Bilge, Ipek Kaplan Bulut, Ayse Pinar Goksu Cetinkaya, Elif Comak, Belde Kasap Demir, Nida Dincel, Osman Donmez, Mehmet Akif Durmus, Hasan Dursun, Ruhan Dusunsel, Ali Duzova, Pelin Ertan, Asuman Gedikbasi, Nilufer Goknar, Sercin Guven, Duygu Hacihamdioglu, Augustina Jankauskiene, Mukaddes Kalyoncu, Salih Kavukcu, Bahriye Uzun Kenan, Nuran Kucuk, Bahar Kural, Mieczysław Litwin, Giovanni Montini, William Morello, Lukasz Obrycki, Beyhan Omer, Ebru Misirli Ozdemir, Nese Ozkayin, Dusan Paripovic, Cemile Pehlivanoglu, Seha Saygili, Franz Schaefer, Susanne Schaefer, Ferah Sonmez, Yilmaz Tabel, Nesrin Tas, Mehmet Tasdemir, Ana Teixeira, Demet Tekcan, Rezan Topaloglu, Sebahat Tulpar, Ozde Nisa Turkkan, Berfin Uysal, Metin Uysalol, Renata Vitkevic, Sevgi Yavuz, Sibel Yel, Tarik Yildirim, Zeynep Yuruk Yildirim, Nurdan Yildiz, Selcuk Yuksel, Eray Yurtseven, Alev Yilmaz

**Affiliations:** ^1^Department of Pediatrics Basic Sciences, Institute of Child Health, Istanbul University, Istanbul, Türkiye; ^2^Division of Pediatric Nephrology, Istanbul Faculty of Medicine, Istanbul University, Istanbul, Türkiye; ^3^Division of Pediatric Nephrology, Centro Materno Infantil do Norte, Centro Hospitalar Universitário do Porto, Porto, Portugal; ^4^Division of Pediatric Nephrology, Faculty of Medicine, Celal Bayar University, Manisa, Türkiye; ^5^Division of Pediatric Nephrology, Faculty of Medicine, Marmara University, Istanbul, Türkiye; ^6^Division of Pediatric Nephrology, Erzurum Training and Research Hospital, University of Health Sciences, Erzurum, Türkiye; ^7^Division of Pediatric Nephrology, Faculty of Medicine, Cukurova University, Adana, Türkiye; ^8^Division of Pediatric Nephrology, Faculty of Medicine, Ondokuz Mayis University, Samsun, Türkiye; ^9^Division of Pediatric Nephrology, Faculty of Medicine, Gazi University, Ankara, Türkiye; ^10^Division of Pediatric Nephrology, Faculty of Medicine, Dokuz Eylul University, Izmir, Türkiye; ^11^Division of Pediatric Nephrology, School of Medicine, Koc University, Istanbul, Türkiye; ^12^Division of Pediatric Nephrology, Faculty of Medicine, Ege University, Izmir, Türkiye; ^13^Division of Pediatric Nephrology, Faculty of Medicine, Akdeniz University, Antalya, Türkiye; ^14^Division of Pediatric Nephrology, Tepecik Training and Research Hospital, University of Health Sciences, Izmir, Türkiye; ^15^Division of Pediatric Nephrology, Izmir Katip Celebi University Faculty of Medicine, Izmir, Türkiye; ^16^Division of Pediatric Nephrology, Dr. Behcet Uz Children Diseases Training and Research Hospital, University of Health Sciences, Izmir, Türkiye; ^17^Division of Pediatric Nephrology, Faculty of Medicine, Uludag University, Bursa, Türkiye; ^18^Department of Medical Microbiology, Istanbul Faculty of Medicine, Istanbul University, Istanbul, Türkiye; ^19^Division of Pediatric Nephrology, Okmeydani Training and Research Hospital, University of Health Sciences, Istanbul, Türkiye; ^20^Division of Pediatric Nephrology, Faculty of Medicine, Erciyes University, Kayseri, Türkiye; ^21^Division of Pediatric Nephrology, Faculty of Medicine, Hacettepe University, Ankara, Türkiye; ^22^Department of Rare Diseases, Institute of Child Health, Istanbul University, Istanbul, Türkiye; ^23^Division of Pediatric Nephrology, Bagcilar Training and Research Hospital, University of Health Sciences, Istanbul, Türkiye; ^24^Division of Pediatric Nephrology, Faculty of Medicine, Bahcesehir University, Istanbul, Türkiye; ^25^Clinic of Pediatrics, Institute of Clinical Medicine, Vilnius University, Vilnius, Lithuania; ^26^Division of Pediatric Nephrology, Faculty of Medicine, Karadeniz Technical University, Trabzon, Türkiye; ^27^Division of Pediatric Nephrology, Kartal Training and Research Hospital, University of Health Sciences, Istanbul, Türkiye; ^28^Department of Pediatrics, Bakirkoy Sadi Konuk Training and Research Hospital, University of Health Sciences, Istanbul, Türkiye; ^29^Division of Nephrology, Kidney Transplantation and Hypertension, The Children’s Memorial Health Institute, Warsaw, Poland; ^30^Division of Pediatric Nephrology, Dialysis and Transplant Unit, Fondazione IRCCS Ca’ Granda - Ospedale Maggiore Policlinico, Milan, Italy; ^31^Department of Clinical Sciences and Community Health, University of Milano, Milan, Italy; ^32^Department of Biochemistry, Istanbul Faculty of Medicine, Istanbul University, Istanbul, Türkiye; ^33^Department of Pediatrics, Okmeydani Training and Research Hospital, University of Health Sciences, Istanbul, Türkiye; ^34^Division of Pediatric Nephrology, Faculty of Medicine, Trakya University, Edirne, Türkiye; ^35^Division of Pediatric Nephrology, University Children’s Hospital, Belgrade, Serbia; ^36^Division of Pediatric Nephrology, Umraniye Training and Research Hospital, Istanbul, Türkiye; ^37^Division of Pediatric Nephrology, Cerrahpasa Faculty of Medicine, Istanbul University-Cerrahpasa, İstanbul, Türkiye; ^38^Division of Pediatric Nephrology, Center for Pediatrics and Adolescent Medicine, Heidelberg University, Heidelberg, Germany; ^39^Division of Pediatric Nephrology, Faculty of Medicine, Adnan Menderes University, Aydin, Türkiye; ^40^Division of Pediatric Nephrology, Faculty of Medicine, Inonu University, Malatya, Türkiye; ^41^Division of Pediatric Nephrology, Faculty of Medicine, Istinye University, Istanbul, Türkiye; ^42^Division of Pediatric Nephrology, Bakirkoy Dr. Sadi Konuk Training and Research Hospital, University of Health Sciences, Istanbul, Türkiye; ^43^Division of Pediatric Nephrology, Dortcelik Children’s Hospital, University of Health Sciences, Bursa, Türkiye; ^44^Division of Pediatric Nephrology, Bursa City Hospital, University of Health Sciences, Bursa, Türkiye; ^45^Division of Pediatric Emergency, Istanbul Faculty of Medicine, Istanbul University, Istanbul, Türkiye; ^46^Division of Pediatric Nephrology, Kanuni Sultan Suleyman Research and Training Hospital, University of Health Sciences, Istanbul, Türkiye; ^47^Department of Pediatrics, Kanuni Sultan Suleyman Research and Training Hospital, University of Health Sciences, Istanbul, Türkiye; ^48^Department of Pediatric Rheumatology and Pediatric Nephrology, School of Medicine, Canakkale Onsekiz Mart University, Canakkale, Türkiye; ^49^Department of Biostatistics, Istanbul Faculty of Medicine, Istanbul University, Istanbul, Türkiye

**Keywords:** children, congenital anomalies of the kidney and urinary tract, CAKUT, heat shock proteins, Hsp70, UTILISE study

## Abstract

**Background:**

Congenital anomalies of the kidney and urinary tract (CAKUT) are defined as structural malformations of the kidney and/or urinary tract. Heat shock proteins (HSPs) are expressed in the kidney in response to cellular changes, such as thermal, hemodynamic, osmotic, inflammatory, and mechanical stresses. This study aimed to assess uHSP70 levels during acute urinary tract infections (UTI) and non-infection periods in patients with CAKUT, and to evaluate whether uHSP70 is elevated in CAKUT subtypes.

**Methods:**

Among patients with CAKUT, 89 patients with UTI (CAKUT-A), 111 without UTI (CAKUT-B), and 74 healthy children were included in the study. uHSP70 levels were measured using enzyme-linked immunosorbent assay (ELISA).

**Results:**

uHSP70 level was significantly higher in the CAKUT-A group than in the CAKUT-B and healthy control groups (p < 0.0001). Moreover, the level of uHSP70 was significantly higher in the CAKUT-B group than in the control group (p < 0.0001), but was not different between the CAKUT subtypes (p > 0.05).

**Conclusion:**

Urine HSP70 can also be used to predict UTI in patients with CAKUT. Moreover, uHSP70 levels were higher in children with CAKUT during the non-infectious period than in healthy controls. This suggests that children with CAKUT are at risk of chronic non-infectious damage.

## 1 Introduction

Congenital anomalies of the kidney and urinary tract (CAKUT) include structural malformations of the kidney and/or the urinary system (1). CAKUT occurs due to disruption of nephrogenesis under the influence of genetic or environmental factors and usually presents as urinary tract dilation on antenatal ultrasound. It has been reported that CAKUT occurs in 1/500 live birth neonates and accounts for 20-30% of all anomalies in the prenatal period (1).

Urinary tract infection (UTI) is a common clinical finding in patients with CAKUT. The UTILISE (Urinary Tract Infection and Levels of heat shock protein 70 In children as a Sensitive marker for Excluding other infections) study showed that urine Heat Shock Protein 70 (uHSP70) is elevated during acute UTI and decreases after antibiotic treatment (2). HSPs are expressed in the kidneys in response to cellular changes, such as thermal, hemodynamic, osmotic, inflammatory, and mechanical stress (3). HSPs increase cell protection and help maintain homeostasis; these stress responses induce HSP production (4). Few studies have investigated the relationship between HSP70 and CAKUT, and most of them address HSP70 in obstructive nephropathy (5, 6). To the best of our knowledge, no study has evaluated uHSP70 expression in other subtypes of CAKUT, such as renal agenesis, multicystic dysplastic kidney, hypo-dysplastic kidney, and/or atrophic kidney. We aimed to assess uHSP70 levels during acute UTI and non-infection periods in patients with CAKUT, and to evaluate whether uHSP70 was elevated in CAKUT subtypes.

## 2 Materials and methods

**Figure 1:**
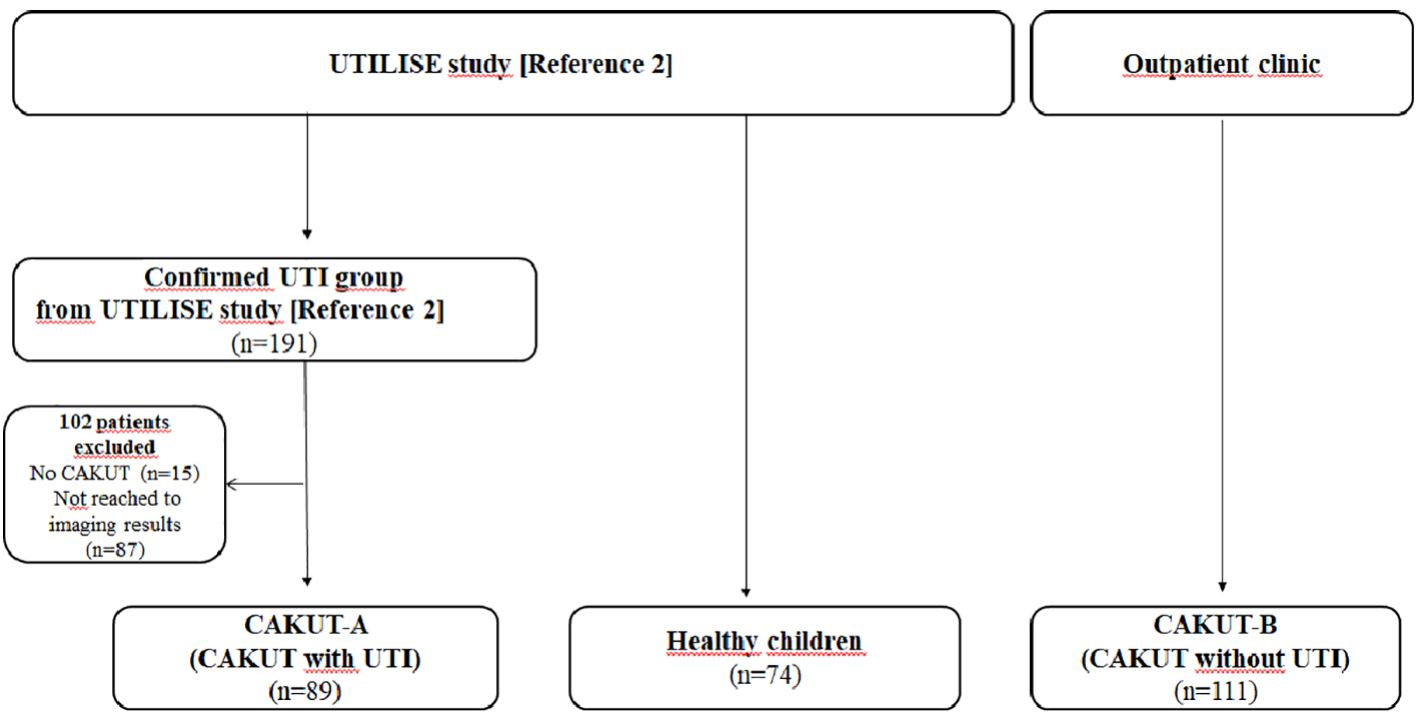
Algorithm for patient selection.

This study was planned as a part of UTILISE study, a prospective multicenter and multinational study (2). The UTILISE study had a group of patients with proven UTI (n=191); among them, we included those with CAKUT in the CAKUT-A group (n=89). The algorithm used for patient selection is shown in **Figure 1**. Patients in this group had symptoms suggestive of UTI and were evaluated using routine physical examination, urinalysis, and urine culture at the time of admission. The sampling method for urine culture was determined by the physician according to the UTI protocol of the participating center. The inclusion criteria for the UTI group were a combination of the following three features: (a) symptoms suggestive of UTI such as fever, dysuria, or abdominal pain; (b) presence of any of positive findings in urinalysis for UTI, such as pyuria, leukocyte esterase positivity or nitrite test; and (c) significant bacterial growth in the urine culture (7). Pyuria is defined as the presence of >5 WBCs per high-power field at the microscopic analysis after centrifugation of the urine (7). Bacterial growth in urine culture was defined as significant if there was growth of ≥ 10^5^ colony forming units (cfu)/mL of a single uropathogen for samples obtained via collecting bag and mid-stream urine, and ≥10^4^ cfu/mL by catheterization (8, 9). All children in the UTI group were treated according to the UTI protocols of the participating centers.

In this study, we included another group of patients with CAKUT, but without UTI (CAKUT-B). The CAKUT-B group consisted of 111 patients recruited from the pediatric nephrology outpatient clinics of the Istanbul Medical Faculty, Umraniye Training and Research Hospital, and Bursa Dortcelik Hospital during the same timeframe as that of the UTILISE study. These patients had no complaints, no leukocyte esterase and/or nitrite positivity on urinalysis, or bacterial growth in urine culture.

The CAKUT-A and CAKUT-B groups comprised patients with renal parenchymal malformations, migration anomalies, and collecting system anomalies. Renal parenchymal malformations include renal agenesis, hypoplasia/dysplasia, and multicystic dysplastic kidney disease. Ectopic and horseshoe kidneys are migratory anomalies. Collecting system anomalies include nonobstructive dilation (NOD) or ureteropelvic junction obstruction (UPJO), ureterovesical junction obstruction (UVJO), megaureter, ureter duplex, ectopic ureter, double collector system, vesicoureteral reflux (VUR), ureterocele, and posterior urethral valve. The imaging and laboratory results of patients in groups A and B were obtained from patient files.

The healthy control group from the UTILISE study (2) was enrolled in this study and consisted of 74 children with no acute and/or chronic diseases or urinary tract abnormalities. In the CAKUT-B and control groups, urine samples were collected midstream and/or in collection bags.

The ethical committee of Istanbul University, Istanbul Faculty of Medicine, approved the study for Turkish centers (2017/752). Ethical approval was obtained from the local Ethics Committees of all other participating countries.

### 2.1 Laboratory analysis

Pre-treatment samples were obtained from the UTI group before the initiation of antibiotic treatment. In the other groups, urine samples were collected once at the time of admission. Random urine samples were obtained and stored at -80°C. Samples from participating centers were sent to the laboratory of the Istanbul University Istanbul Faculty of Medicine on dry ice. Urine samples that were stored at -80°C were brought to room temperature before the analysis. uHSP70 levels were measured using enzyme-linked immunosorbent assay (ELISA) using a HSP70 ELISA Kit (Cat no: KTE62748) purchased from Abbkine (Abbkine, Inc., China), following the manufacturer’s instructions. The levels of HSP70 were expressed as ng/mL. The detection and quantification limits were set to < 0.05 ng/ml for HSP70. The intra-assay coefficient of variation (CV) for HSP70 were 7.9% and 8.6%.

### 2.2 Statistical analysis

All statistical analyses were performed using the Statistical Package for Social Sciences (SPSS) for Windows, version 22.0 (IBM Corp., Armonk, NY, USA). Data for continuous variables are presented as percentages or medians and interquartile ranges (IQR). The normality of the parameter distribution was tested using the Kolmogorov-Smirnov test. Non-parametric tests (Mann-Whitney U test or Kruskal-Wallis test in cases of more than two groups) were used for between-group comparisons. The Mann-Whitney U test was used to test the significance of pairwise differences, with Bonferroni correction applied to adjust for multiple comparisons. When investigating changes in uHSP70 levels between groups, age effects were adjusted using analysis of covariance (ANCOVA). For all statistical analyses p < 0.05 was considered statistically significant.

## 3 Results

Totally 89 CAKUT patients with UTI (CAKUT-A), 111 CAKUT patients without UTI (CAKUT-B), and 74 healthy children were included in this study. Sex distribution was comparable in all groups (p=0.071). Patients in the CAKUT-A group were younger than those in the other groups (p < 0.0001) (**Table 1**). uHSP70 median level was not different between the children with estimated glomerular filtration rate (eGFR) ≥90 and <90 mL/min/1.73m^2^ (p>0.05).

**Table 1:** Demographic data of the study and control groups and uHSP70 levels.

	CAKUT-A group (n=89)	CAKUT-B group (n=111)	Control group (n=74)	p
**Age (years)**	5.0 (1.4-8.5)	7.4 (4.0-11.5)	7.6 (4.9-10.4)	**<0.0001**
**Gender** Girls n (%)	60 (67)	57 (51)	42 (57)	0.071
**uHSP70 (ng/mL)**	139.6 (62.1-188.5)	49.3 (39.6-58.3)	32.8 (29.4-40.1)	**<0.0001**

Data are given as percent or median (interquartile rage) as appropriate. CAKUT, Congenital Anomalies of the Kidney and Urinary Tract; uHSP70, urine Heat Shock Protein 70.

**Figure 2:**
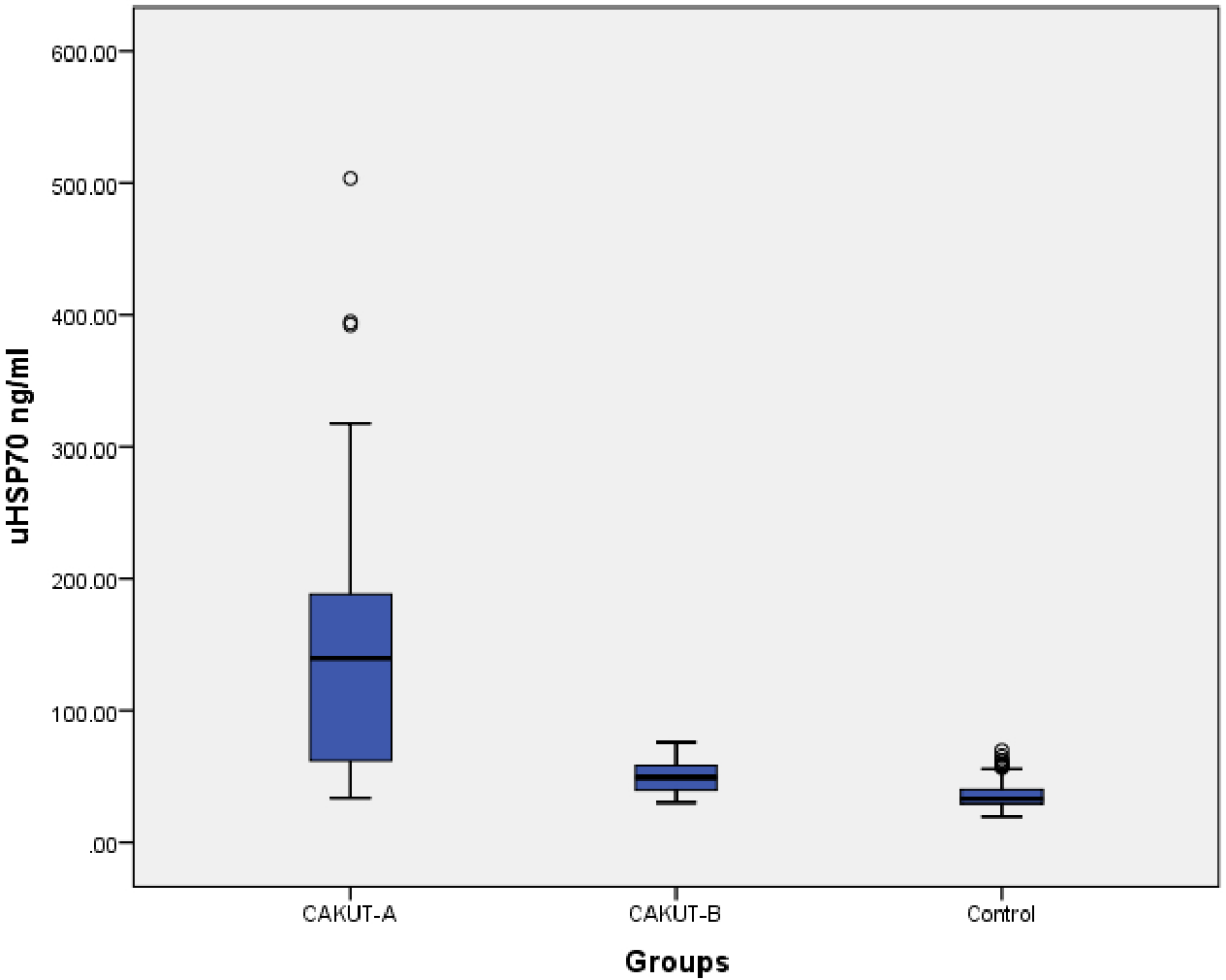
Comparison of the urine HSP70 levels between CAKUT-A, CAKUT-B, and Control groups.

uHSP70 levels were significantly higher in the CAKUT-A group than in the CAKUT-B and healthy control groups (p < 0.0001) (**Table 1**). When the effect of age was adjusted using covariance analysis, uHSP70 levels were higher in the CAKUT-A group than in the other two groups (p < 0.0001). Furthermore, uHSP70 expression in the CAKUT-B group was significantly lower than that in the CAKUT-A group (p < 0.0001), whereas it was significantly higher than that in the control groups (p < 0.0001) (**Table 1**; **Figure 2**).

### 3.1 Comparison of CAKUT subtypes

The CAKUT subtypes are listed in **Table 2**. In the CAKUT-A group, collecting system anomalies occurred in 91% (n=81) of the patients, of whom 55% were diagnosed with VUR. In this group, nonobstructive dilation was the second most frequent disease, with a ratio of 30%. Moreover, some patients with VUR in the CAKUT-A group were also diagnosed with UVJO (n=2), UPJO (n=1) and ureterocele (n=1).

**Table 2:** Identification of CAKUT subtypes.

	CAKUT-A group (n=89)	CAKUT-B group (n=111)
**Collecting system anomalies (n)** Vesicoureteral reflux n (%) Non-obstructive hydronephrosis n (%) Ureteropelvic junction obstruction (postop) n (%) Ureterovesical junction obstruction (postop) n (%) Duplex system n (%) **Renal parenchymal malformations (n)** Unilateral renal agenesis n (%) Renal hypo-dysplasia n (%) Multicystic dysplastic kidney n (%) Atrophic kidney n (%) **Migration anomalies (n)** Ectopic kidney n (%) Horseshoe kidney n (%) **Total**	**81** 49 (55) 27 (30) 4 (5) 1 (1) 0 **7** 2 (2) 5 (6) 0 0 **1** 1 (1) 0 **89 (100)**	**48** 40 (36) 6 (5) 0 1 (1) 1 (1) **56** 24 (22) 12 (11) 15 (14) 5 (5) **7** 6 (5) 1 (1) **111 (100)**

CAKUT, Congenital Anomalies of the Kidney and Urinary Tract.

**Figure 3:**
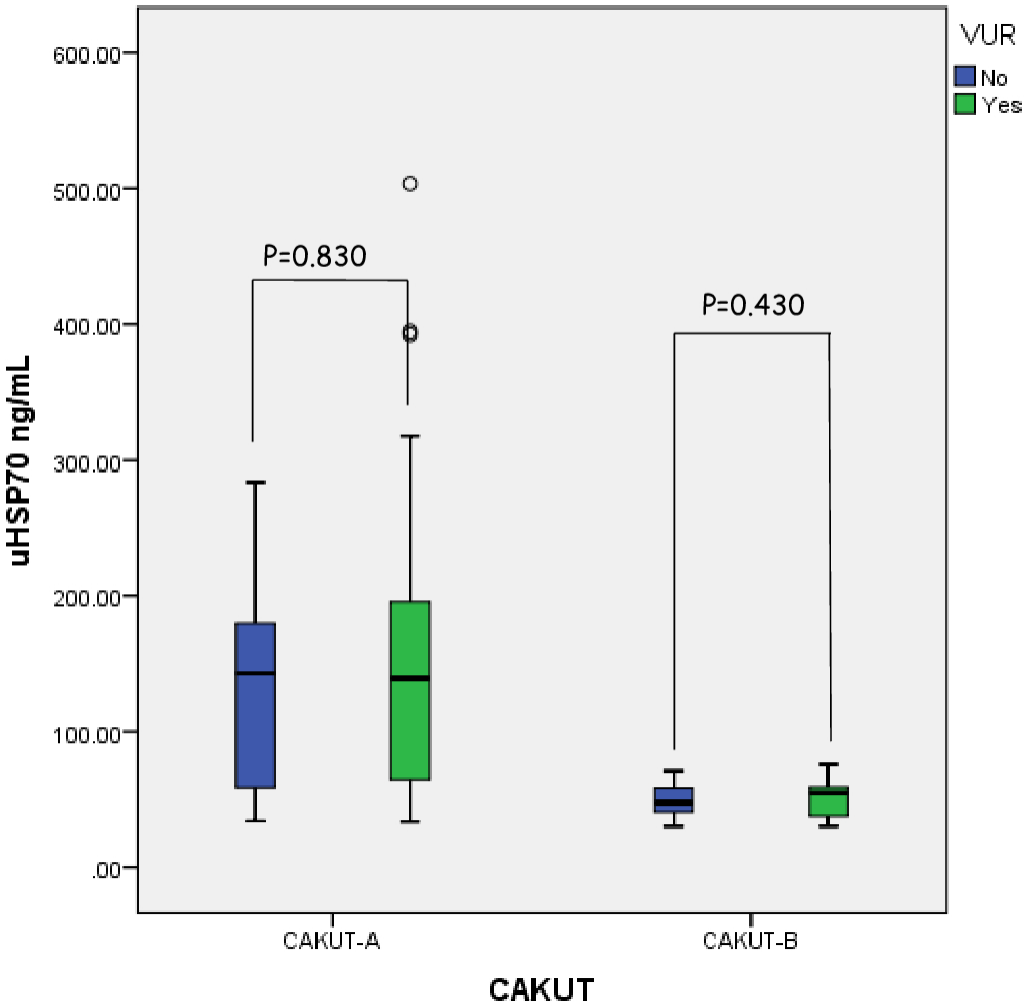
Comparison of the urine HSP70 levels between the patients with VUR in CAKUT-A and CAKUT-B.

When we compared CAKUT-A patients with VUR and without VUR, uHSP70 median levels were not different between these two groups (139.1 ng/mL and 142.9 ng/mL, respectively; p=0.830) (**Figure 3**).

Renal parenchymal malformations and collecting system anomalies constituted 52% and 43% of the CAKUT-B group, respectively. In this group, seven patients with VUR had additional anomalies [posterior urethral valve (n=3), duplex system (n=3), and meningomyelocele (n=1)]. When we analyzed the subgroups of CAKUT-B, there was no difference in median HSP70 levels between collecting system anomalies and renal parenchymal malformations (49.9 ng/mL vs. 49.0 ng/mL; p=0.940). Collecting system anomalies and renal parenchymal malformations in the CAKUT-B group were compared separately with the control group. Median uHSP70 levels were higher in patients with collecting system anomalies (49.9 ng/mL vs. 32.8 ng/mL; p < 0.0001) and renal parenchymal disorders (49.0 ng/mL vs. 32.8 ng/mL; p < 0.0001) compared to controls. Median uHSP70 levels did not differ between patients with and without VUR in the CAKUT-B group (55.3 ng/mL vs. 47.5 ng/mL; p=0.434). A comparison of the uHSP70 levels between patients with and without VUR in the CAKUT-A and CAKUT-B groups was shown in **Figure 3**.

## 4 Discussion

In our previous UTILISE study, we evaluated children with UTI and found increased uHSP70 levels during infection (2). The presence of CAKUT is one of the most important risk factors for developing UTI (10). Our results in current study showed that uHSP70 level was significantly higher in both CAKUT-A and CAKUT-B groups than in the control group. In addition, the uHSP70 levels were significantly higher in the CAKUT-A group than in the CAKUT-B group. It was expected that uHSP70 is at its highest level during the UTI period, as the UTILISE study clearly demonstrated that uHSP70 is a reliable biomarker for predicting UTI. Further, uHSP70 is significantly higher during infection in patients with CAKUT, suggesting that uHSP70 levels could be used to predict UTI in patients with CAKUT. Interestingly, the level of uHSP70 in the non-infectious period was higher than that in the controls but much lower than that in the infectious period. We believe that the increased uHSP70 levels in the CAKUT-B group may be due to ongoing oxidative stress during the chronic process in patients with CAKUT. CAKUT is a chronic disease that meets the definition of chronic kidney disease (CKD) with evidence of kidney damage for 3 months or more as demonstrated by the presence of structural kidney disorders and by imaging methods (11). Many studies have shown that oxidative stress is increased in children with CKD (12, 13). In addition, it should be kept in mind that previous UTI attacks may contribute to this situation. We have earlier reported that uHSP70 was slightly elevated in patients with kidney-related chronic diseases, such as CKD, IgA nephropathy, and diabetic nephropathy (14, 15). We also showed that uHSP70 levels were excessively elevated during UTI, an acute disease, and decreased in response to antibiotic treatment. The presence of CAKUT did not preclude the use of uHSP70 as a biomarker for UTI.

An experimental study in rats showed that HSP70 levels increased after 10 days of unilateral obstruction in UPJ. After the obstruction was resolved, HSP70 levels returned to baseline. This suggests that HSP70 levels may be a specific and localized response to oxidative injury (16). Valles et al. (6) evaluated 22 children with congenital UPJO. Renal biopsies were obtained for immunohistochemical and western blot analyses at the time of surgery. Increased staining of HSP27 and HSP70 in the proximal tubules, cortical collecting ducts and medullary collecting ducts was detected using immunohistochemical method. This indicates that HSP27 and HSP70 could play a role in the adaptive response of the kidney to congenital UPJO (6). Oktar et al. (5) investigated the uHSP70 levels in 43 children with UPJO who underwent pyeloplasty, 25 patients with NOD, and 30 healthy children. The uHSP70 levels were higher in the pyeloplasty group than in the NOD and control groups. Additionally, the uHSP70 levels decreased postoperatively. It has been emphasized that uHSP70 can predict surgical indications for unilateral UPJO (5). Moreover, adult UPJO rats treated with α-tocopherol, a compound used to treat acute oxidative injury, showed decreased HSP70 expression in kidneys (17). In previous studies, the elevation of HSP70 in obstructive uropathy was explained by the increased intrarenal oxidative stress in UPJO, which includes kidney injury, and the increased synthesis of HSP70 from injured renal cells in response to this stress (18–20).

Because the number of patients with obstructive uropathy in our study was small (only 12 patients), we did not compare patients with obstructive uropathy to those with other subtypes of CAKUT according to uHSP70 levels. In our study, uHSP70 was evaluated separately according to the subtypes of CAKUT in every CAKUT group, including VUR, collecting system anomalies, and renal parenchymal malformations. Urine HSP70 levels were significantly higher in all CAKUT subtypes; we therefore concluded that uHSP70 levels were not specific to any of the CAKUT subgroups.

However, this study has some limitations. The number of patients in the CAKUT subgroups was not evenly distributed; therefore, we did not analyze the statistical significance between them.

## 5 Conclusions

Urine HSP70 levels in CAKUT patients were significantly high while they were diagnosed with UTI. uHSP70 levels can be used to predict UTI in patients with CAKUT. Moreover, uHSP70 levels were higher in children with CAKUT during the non-infectious period than in healthy controls. This suggests that children with CAKUT are at risk of chronic non-infectious damage.

## Data availability statement

The original contributions presented in the study are included in the article/supplementary material. Further inquiries can be directed to the corresponding author.

## Ethics statement

The studies involving humans were approved by The ethical committee of Istanbul University Istanbul Faculty of Medicine approved the study for Turkish centers (2017/752). The studies were conducted in accordance with the local legislation and institutional requirements. Written informed consent for participation in this study was provided by the participants’ legal guardians/next of kin.

## Author contributions

BAk: Conceptualization, Data curation, Formal analysis, Funding acquisition, Investigation, Methodology, Project administration, Resources, Software, Supervision, Validation, Visualization, Writing – original draft, Writing – review & editing. AA, IA, HA: Conceptualization, Data curation, Formal analysis, Investigation, Methodology, Resources, Validation, Writing – review & editing. BAt, OA: Conceptualization, Data curation, Formal analysis, Investigation, Methodology, Resources, Writing – review & editing. SB, AB, MB, IB: Conceptualization, Data curation, Formal analysis, Investigation, Methodology, Resources, Writing – review & editing. IKB: Conceptualization, Data curation, Formal analysis, Investigation, Methodology, Writing – review & editing. AC, EC, BD, ND, OD, MD, HD, RD, AD: Conceptualization, Data curation, Formal analysis, Investigation, Methodology, Resources, Writing – review & editing. PE: Conceptualization, Data curation, Formal analysis, Investigation, Methodology, Resources, Writing – review & editing. AG: Conceptualization, Data curation, Formal analysis, Investigation, Methodology, Resources, Writing – review & editing. NG, SG, DH, AJ, MK, SK, BUK, NK, BK, ML, GM, WM, LO, BO, EMO, NO, DP, CP, SSa, FSc SSc, FSo, YT, NT, MT, AT: Conceptualization, Data curation, Formal analysis, Investigation, Methodology, Resources, Writing – review & editing. DT: Conceptualization, Data curation, Investigation, Methodology, Writing – review & editing. RT, ST, OT, BU, MU, RV, Sya, Sye, TY: Conceptualization, Data curation, Formal analysis, Investigation, Methodology, Resources, Writing – review & editing. ZY: Conceptualization, Data curation, Formal analysis, Funding acquisition, Investigation, Methodology, Project administration, Resources, Software, Supervision, Validation, Visualization, Writing – original draft, Writing – review & editing. NY, SYu, EY: Conceptualization, Data curation, Formal analysis, Investigation, Methodology, Resources, Writing – review & editing. AY: Conceptualization, Data curation, Formal analysis, Funding acquisition, Investigation, Methodology, Project administration, Resources, Software, Supervision, Validation, Visualization, Writing – original draft, Writing – review & editing.
